# Retraction: Construction and characterization of a magnetic nanoparticle-supported Cu complex: a stable and active nanocatalyst for synthesis of heteroaryl-aryl and di-heteroaryl sulfides

**DOI:** 10.1039/d5ra90060c

**Published:** 2025-05-15

**Authors:** Yutong Fang, Songlin Chen, Li-Yuan Chang

**Affiliations:** a Sinopec Research Institute of Petroleum Processing Beijing 100089 China; b Department of Basics, Naval University of Engineering Wuhan 430030 Hubei China chensonglin0170@163.com; c School of Resource and Environmental Engineering, Wuhan University of Science and Technology Wuhan 430070 Hubei China; d Institute of Chemical and Nanotechnology Research Shanghai China liyuanchang839@gmail.com

## Abstract

Retraction of ‘Construction and characterization of a magnetic nanoparticle-supported Cu complex: a stable and active nanocatalyst for synthesis of heteroaryl-aryl and di-heteroaryl sulfides’ by Yutong Fang *et al.*, *RSC Adv.*, 2024, **14**, 812–830, https://doi.org/10.1039/D3RA07791H.

The Royal Society of Chemistry hereby wholly retracts this *RSC Advances* article due to concerns with the reliability of the data.

Multiple NMR spectra show signs of manipulation. The authors were contacted but did not provide a response to the concerns.

Given the significance of these concerns, the Editor has lost confidence that the findings presented in this paper are reliable.

The authors were informed about the retraction of the article but have not responded.

Signed: Laura Fisher, Executive Editor, *RSC Advances*

Date: 9th May 2025

